# Inflammatory gene expression signatures in idiopathic intracranial hypertension: possible implications in microgravity-induced ICP elevation

**DOI:** 10.1038/s41526-017-0036-6

**Published:** 2018-01-11

**Authors:** Susana B. Zanello, Vasisht Tadigotla, James Hurley, Johan Skog, Brian Stevens, Eusebia Calvillo, Eric Bershad

**Affiliations:** 1 KBRwyle, NASA Johnson Space Center, Houston, TX USA; 2 Exosome Diagnostics, Cambridge, MA USA; 3 Baylor College of Medicine, Houston, TX USA

## Abstract

The visual impairment and intracranial pressure (VIIP) syndrome is a neuro–ophthalmologic condition described in astronauts returning from long duration space missions. Idiopathic intracranial hypertension (IIH), also known as pseudotumor cerebri, is characterized by a chronic elevation of intracranial pressure (ICP) in the absence of an intracranial mass lesion. Because VIIP and IIH share some neurologic and ophthalmologic manifestations, the latter might be used as a model to study some of the processes underlying VIIP. This work constitutes a preliminary investigation of the molecular pathways associated with the elevation of ICP in IIH. Gene expression signatures were obtained from exosomes collected from CSF and plasma in patients with possible signs of IIH. The gene expression targets focused on inflammatory genes and miRNAs. The results suggest that inflammatory cytokine-driven processes and immune cell migration are activated when ICP is elevated in IIH patients, either as a cause or effect of the ICP increase. Several miRNAs appear to be involved in this response, among which miR-9 and miR-16 are upregulated in CSF and plasma of higher ICP subjects. This study provides evidence in support of neurophysiological alterations and neuro-immunomodulation in this condition. If similar changes are seen in astronauts manifesting with the VIIP syndrome, an underlying pathophysiological basis may be discovered.

## Vision: Brain inflammation could explain astronauts’ visual impairments

People with elevated pressure inside their skulls show signs of inflammation that could be relevant for astronauts on long-duration missions. To better understand the biology of spaceflight-induced visual impairment—a common problem for astronauts that is thought to arise from pressure building up around the brain in microgravity—a team led by Susana Zanello from the NASA Johnson Space Center in Houston, Texas, USA, examined the spinal fluid and blood of individuals with idiopathic intracranial hypertension, a neurologic condition that shares some features with the mysterious syndrome that impairs astronauts’ sight vision. The researchers characterized the RNA contained within tiny secreted particles, known as exosomes, in the patient samples. The expression of several genes and regulatory microRNAs linked to pro-inflammatory immune responses were changed. The authors surmise that similar immune changes could underpin the eyesight syndrome plaguing astronauts.

## Introduction

Human space exploration involves multi-system health risks. Neuro-ophthalmologic symptoms, including elevated intracranial pressure (ICP) upon return to Earth,^1^ have been observed in astronauts participating in long-duration missions, a condition named visual impairment and intracranial pressure syndrome (VIIP). On Earth, these neuroanatomical findings concur with those in idiopathic intracranial hypertension (IIH).^2^ The etiology of IIH is a topic of debate. The moderately elevated ICP over many years has been linked to cognition losses relieved by lumbar tapping to reduce ICP.^3,4^ While most cases are seen in overweight women of child-bearing age, there is no specific condition on Earth that shares more similarities to VIIP, constituting a reasonable analog to study the effects of chronically elevated ICP at the molecular level.

A number of performance^5–7^ and neurologic signs (“space fog”)^8^ have also been linked to spaceflight and might also result from elevation of ICP. In this paper, we test the hypothesis that these symptoms are caused by disturbances in the neurophysiology of the brain and correlated with molecular markers in the cerebrospinal fluid (CSF) by studying gene expression profiles from CSF and plasma in individuals with suspected IIH. The gene expression signatures found suggest a systemic inflammatory status corresponded by local brain inflammatory processes in subjects with elevated ICP. Putative biomarker candidates are discussed, although further studies are necessary to validate their possible research and clinical use. These findings may have implications in understanding neurophysiologic changes during spaceflight.

## Results

### Subject information

Patient information is presented in Table 1 and the Supplementary Table e-1. The average age was 33.8 ± 9.7 years (range 18–51), 2 males and 20 females. No subject within the lower ICP group was diagnosed with IIH. The subjects in the higher ICP group were diagnosed with IIH and exhibited typical ocular symptoms. No correlation was found between ICP and RNFL thickness (Pearson correlation coefficient between ICP and the average RNFL of both eyes was less than 0.4).

**Table 1:** Subject distribution summarizing the mean ICP and standard deviation, ICP range and retinal nerve fiber layer (RNFL) measured by optical coherence tomography (OCT) and averaged between the two eyes on each individual

	Lower ICP group	Higher ICP group
*N*	7	14
Mean ICP	14.6 mmHg ± 2.5	24.0 mmHg ± 4.1
ICP range	11.0–17.9 mmHg	18.8–31.4 mmHg
Mean RNFL	147.6 µm ± 119.9	195.2 µm ± 116.3

Of 22 subjects recruited, ICP was not able to be determined in one of them due to technically difficult LP. That subject was therefore not included in the study and data collection was completed in 21 subjects. The cut off between normal and elevated ICP was established at 18 mm Hg

### miRNA and mRNA expression in higher and lower ICP subjects

In plasma, the average detection rate reached almost 45% of the targets in the TaqMan Open Array miRNA panel and 75% for the mRNA Inflammation Open Array. In CSF, the average detection rate for miRNAs was approximately 15% of the targets and approximately 25% for mRNA targets. Very few samples (two or less) showed a lower detection rate and those were excluded from analysis. Table 2 lists the differentially expressed miRNAs and mRNAs between subjects in the higher and lower ICP groups, after a Mann–Whitney test was applied with *p* < 0.05 (FDR not applied). The sensitivity and reproducibility of the mRNA assay was very high as evaluated by multiple extractions and qRT-PCRs (*R*^2^ > 0.95 and 0.9 in plasma and CSF samples, respectively).

**Table 2:** Differentially expressed genes between subjects with normal-to-mildly elevated ICP and with ICP higher than 18 mmHg

Plasma				
miRNA	*U* value	*p* value	Ratio (higher ICP/lower ICP)	Fold change (log2 ratio)
dme-miR-7	80	0.007	29.53	4.88
hsa-miR-938	78.5	0.009	48.11	5.59
hsa-miR-143	76.5	0.013	579.88	9.18
hsa-let-7i	77	0.013	16.73	4.06
hsa-miR-618	14.5	0.014	0.009	−6.80
hsa-miR-374	76	0.015	78.96	6.30
hsa-miR-657	15	0.017	0.07	−3.84
hsa-miR-190b	15	0.017	0.003	−8.38
hsa-miR-10b	16	0.021	0.18	−2.47
hsa-miR-589	74	0.025	16.38	4.03
hsa-miR-16-1	74	0.026	20.45	4.35
hsa-miR-298	18.5	0.027	0.0001	−13.29
hsa-miR-551b	73	0.029	594.24	9.21
Hsa-miR-545	73	0.031	17.08	4.09
Hsa-miR-375	19	0.038	0.003	−8.38
Hsa-miR-10b	19	0.039	0.29	−1.79
Hsa-miR-145	71.5	0.042	30.74	4.94
Hsa-miR-483-5p	19.5	0.043	0.4	−1.32
Hsa-let-7a	70.5	0.044	42.02	5.39
Mmu-miR-187	71	0.045	31.91	5.00
Hsa-miR-154	20.5	0.045	0.008	−6.97
Gene target				
CXCR3	10	0.0008	0.05	−4.32
TLR3	13	0.016	0.19	−2.39
LEFTY2	70	0.02	2.64	1.4
TNFSF4	67.5	0.035	6.01	2.59
CD70	17	0.038	0.12	−3.06
EREG	67	0.038	51.93	5.70
CD80	17	0.038	0.17	−2.56

CSF				
miRNA	*U* value	*p* value	Ratio (higher ICP/lower ICP)	Fold change (log2 ratio)
hsa-miR-9	75.5	0.018	24.89	4.64
hsa-miR-16	71	0.045	25.89	4.69
Gene target				
STAT5B	70.5	0.006	106.98	6.74
TGM2	67.5	0.011	58.04	5.86
CD86 (CD80)	12.5	0.018	0.03	−5.06
S100A9	62.5	0.036	91.53	6.52
RIPK2	16	0.048	0.11	3.18

The differentially expressed miRNAs and mRNAs with *p* < 0.05 as determined by the Mann–Whitney test are shown for both plasma and CSF samples. Ratio values that are <1 indicate downregulation. The list can be further filtered for *U* values lesser than the *U* critical value (20 for plasma and 22 for CSF), providing more stringency

### Pathway analysis

**Fig. 1:**
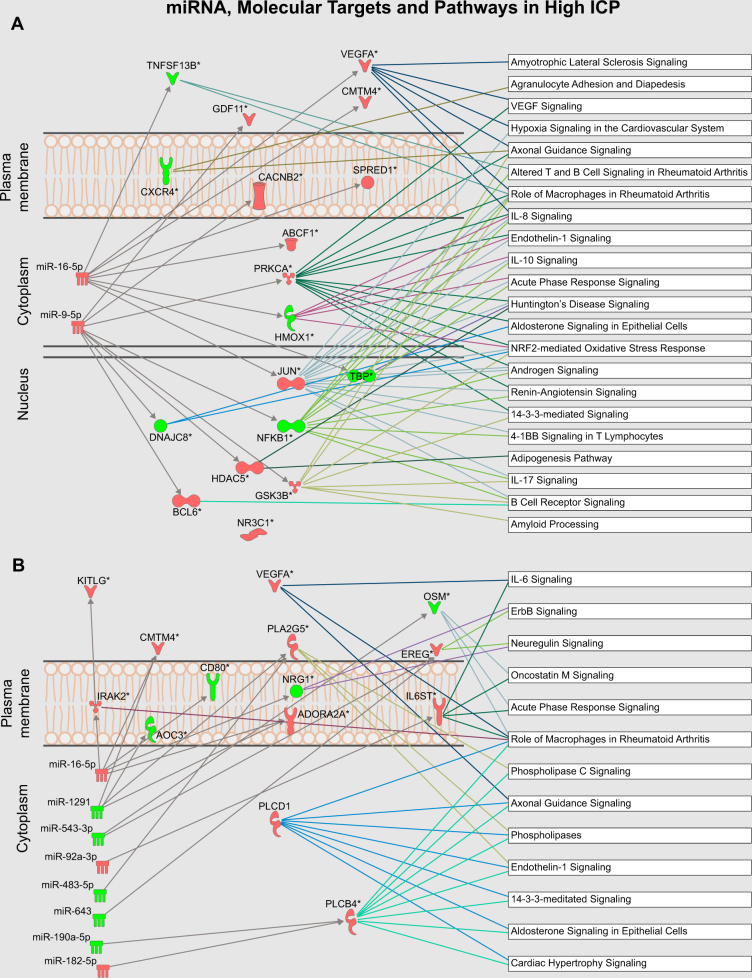
Main miRNA and canonical pathways represented in patients with elevated ICP. miRNA Target Filter Analysis (Ingenuity Pathways Analysis, IPA^®^, QIAGEN Redwood City, www.qiagen.com/ingenuity) was applied to the differentially expressed gene data set from CSF (**a**) and plasma (**b**). miRNA and target relationships are shown by arrows, as well as the main possibly affected canonical pathways

miRNA Target Filter analysis in IPA was performed for the complete gene expression data from the miRNA and Inflammation mRNA panels in CSF and plasma. The process allows the combination of experimental gene expression data with the existing knowledge base of predicted and validated miRNA-target relationships. Figure 1 summarizes the result of this process, depicting miRNAs, their targets and the most represented canonical pathways related to inflammation, eicosanoid–phospholipase signaling and immune cell migration.

A comparison was run between the CSF and plasma expression data sets. Of 205 genes with expression data in plasma and 162 genes in CSF, only 31 were represented in both, and only two, miRNA-16 and CD80 (CD86), were differentially expressed in both CSF and plasma (Table 2). miR-9 and miR-16 were upregulated by approximately 4.7 fold in CSF and plasma of higher ICP subjects, and CD80/86 was downregulated by −5.0 fold and −2.5 fold in CSF and plasma, respectively.

## Discussion

This paper reports a gene expression survey of exosomal RNA in CSF and plasma from patients with elevated ICP (>18 mmHg) compared to patients with normal to moderately elevated ICP (11–18 mmHg). All subjects recruited for the study reported to the neurology clinic, therefore none represented a completely “healthy” volunteer and results need to take into account the existence of underlying conditions.

Although this study only examined a limited panel of mRNAs and miRNAs, it is expected that a similar proportion of overlapping genes would have been found with a wider expression screening, like whole genome microarray or RNAseq. In spite of the small overlap in the set of differentially expressed genes from CSF and plasma, the identified pathways associated with increased ICP were similar. Pro-inflammatory pathways such as acute phase response and interleukin signaling were highly represented. Both gene sets suggest that inflammatory processes are acting both locally in the brain as well as systemically, perhaps contributing to IIH pathophysiology.

Pathway analysis at the CSF level also suggested processes of brain cell death, possibly targeted by miR-9. This hypothesis is also supported by evidence that involves miRNA-9 in degenerative diseases,^9^ as well as promoting glial activation via the NFkB pathway.^10^ Interestingly, miR-9 is itself targeted by miR-16, which was also observed to be upregulated in the high ICP group. Circulating miRNAs are increasingly gaining attention as screening biomarkers in central nervous system (CNS) diseases.^11^ In this work, identified candidates warrant monitoring in larger cohort studies to investigate their biomarker value, i.e., miR-16, also investigated as a biomarker for glioblastoma,^12^ and CD86 (CD80), which has been implicated in progressive inflammatory myelopathy.^13^

For a long time, the CNS has been considered a site of immune privilege, suffering from costly physiologic consequences if subject to inflammation due to trauma or infection. Currently, this paradigm is being reexamined by work highlighting the protective action that a controlled inflammatory response may have on the CNS. For example, it has been shown that mice deficient in functional T cells underperform normal mice in cognitive tasks and this pro-cognitive effect may be mediated by IL-4.^14,15^ Our work supports the existence of an underlying subclinical inflammatory status in IIH. Further studies should be directed towards assessing the extent of these processes and their correlation with neurocognitive parameters.

This study has some limitations. First, it constitutes an exploratory study with small sample size. Second, the lower ICP average, 14.6 mmHg, lies on the higher limit of what is considered a normal ICP (5–15 mmHg), and therefore the comparison with high ICP may not yield as many differentially expressed genes as would be expected in comparison with a normal ICP group. Third, IIH patients are arguably a distinct population from the normal healthy astronaut. Since IIH is more prevalent in women, this study contains a majority of females, also deviating from the sample population of astronauts so far screened for VIIP.

Our findings support new concepts regarding the communication between the immune and nervous systems. The IIH patients evidenced signs of generalized inflammation that may be affecting neural physiology at various levels, from neural signaling to cognition.^16^ These patients were predominantly obese (average BMI = 34.7 ± 7.4 kg/m^2^), with likely associated metabolic syndrome, chronic low-grade inflammation, and insulin resistance and other obesity-associated complications, for which pathways and processes were identified in the analysis.

The value of this study predominantly lies in being the first evaluation of the molecular players in IIH and the first comparison between expression profiles from exosomes in CSF and plasma for this condition. Because elevation of ICP is a hallmark of the disease, findings from this study may be relevant to the VIIP syndrome. The results from the CSF survey suggest neuro–immunomodulatory processes, and further work including astronauts exposed to microgravity and the concomitant fluid shifts with ICP increase, is proposed to test this hypothesis on a more relevant sample population.

## Methods

### Sample collection

Methods were performed in accordance with relevant regulations and guidelines. The study was reviewed and approved by the institutional review boards at Johnson Space Center and Baylor College of Medicine (BCM), Houston, TX. Subject recruitment was done at BCM and written informed consent was obtained from all subjects.

The study population was a pool of neurological patients with suspected IIH, requiring diagnostic or therapeutic LP. Subjects were recruited on the basis of the modified Dandy criteria for IIH and the IIH treatment trial,^17^ consisting in the presence of signs and symptoms of raised ICP (>20 cm H_2_O = 14.7 mmHg), absence of localized findings except those from increased ICP, or abnormal neuroimaging except for empty sella turcica and distended optic nerve sheath. The subject was awake and alert, and no other cause of increased ICP was present. For the LP, the subjects lay in the lateral decubitus position with legs slightly extended. Once the needle was in the thecal sac at the L3-L4 or L4-L5 region, the manometer was observed for the presence of pulse and respiratory waves to indicate patent communication between needle and subarachnoid space. Next, the opening pressure was monitored over a period of 5 min, ICP was measured every minute, and the average calculated. CSF was then drained as per the normal clinical procedure. A volume of 5 ml was collected specifically for the purpose of this study.

Of 22 subjects recruited, ICP was not able to be determined in one of them due to technically difficult LP. That subject was therefore not included in the study and data collection was completed in 21 subjects. The cut off between normal and elevated ICP was established at 18 mm Hg. Patients were grouped into “normal to moderately high ICP” subjects (less than 18 mmHg) and high ICP (higher than 18 mmHg). Seven “normal to mild ICP” and 14 high ICP subjects were compared in the study.

Additionally, subjects had 10 ml blood drawn for plasma separation in K_2_EDTA BD Vacutainer^®^ blood collection tubes with gel barrier. All plasma and CSF samples were pre-filtered through a 0.8-µm syringe filter prior to microvesicular processing, aliquoted and stored at −80 °C in a freezer at the Center for Space Medicine at BCM and then shipped to the Exosome Diagnostics facilities in Cambridge, MA.

### Exosome and RNA isolation

CSF at volume of 5 ml and plasma at volume of 4 ml (pre-filtered) were used for exosome isolation by the ExosomeDx spin column-based method (expRNAeasy SerumPlasma Maxi kit, Qiagen, Valencia, CA). The method allows the reproducible isolation of high-quality exosome-specific RNA, which includes both mRNA and miRNA fractions. Briefly, the pre-filtered sample was mixed 1:1 with 2× binding buffer (XBP) and added to the exoEasy membrane affinity column to bind the exosomes to the membrane. After centrifugation, the flow-through was discarded and wash buffer (XWP) was added to the column to wash off non-specifically retained material. After another centrifugation and discarding of the flow-through, the vesicles were lysed by adding QIAzol solution and following the addition of chloroform, thorough mixing and centrifugation to separate organic and aqueous phases, the aqueous phase was recovered and mixed with ethanol. The sample–ethanol mixture was added to an RNAeasy MinElute spin column and centrifuged. The column was washed once with buffer RWT, and then twice with buffer RPE followed by elution of RNA in a final volume of 14 μL water.

### MicroRNA analysis

Reverse transcription (RT) of miRNA was performed using the Megaplex™ RT Primers (ThermoFisher Scientific, Waltham, MA) to prepare cDNA for real-time PCR analysis on a TaqMan^®^ MicroRNA Array, including a pre-amplification step. Collectively, the Human Pool Set v3.0 containing two Megaplex™ RT Primer Pools, Pools A v2.1 and B v3.0 cover 754 unique microRNAs. The RT reaction for each primer pool was set as follows: 1× Megaplex RT Primers (Pool A or B), 2 mM dNTPs (with dTTP), 10 U/μL MultiScribe reverse transcriptase, 1.5 mM MgCl2, 1× RT buffer, 0.25 U/ μL RNAse inhibitor, in a final volume of 15 μL per reaction. The reaction was run through the temperature sequence per the manufacturer’s instructions.

Preamplification was performed in a 50 μL reaction with a 10 μL aliquot of the RT reaction as a template, 1× TaqMan^®^ PreAmp Master Mix and 1× Megaplex™ PreAmp Primers (Pool A or Pool B), in a temperature cycling (16 cycles) according to the manufacturer. Pre-amplified samples were diluted 1:10 in 0.1× TE pH 8.0. The TaqMan^®^ OpenArray^®^ Human MicroRNA Panel was used for final PCR detection.

In this final step, the DNA polymerase of the TaqMan Universal PCR Master Mix amplifies the specific cDNA targets using sequence-specific primers and probe on the TaqMan microRNA Array. 25 µL TaqMan^®^ OpenArray^®^ Real-Time PCR Master Mix and 13 µL 0.1× buffer TE pH 8.0 were loaded into each of two adjacent wells per sample on a clean 96-well plate. For each sample, 12 µL of diluted Pool A PreAmp was pipetted into one well of each pair and 12 µL of diluted Pool B PreAmp was pipetted into the other well for a total volume of 50 µL in each well. For each sample and primer pool set (Pool A or Pool B), 5 µL of each reaction mixture was pipetted into each of eight wells on an OpenArray 384-well sample plate. Samples were loaded onto OpenArray^®^ plates using the standard Accufill™ protocol. Amplification was performed according to the protocol established for the TaqMan^®^ OpenArray^®^ Human MicroRNA panel.

### mRNA analysis

The RT reaction was carried out in a final volume of 20 μL containing: 14 μL RNA sample, 2 μL Superscript reverse transcriptase enzyme mix, and 4 μL VILO Master Mix (which includesRNaseOUT™ Recombinant Ribonuclease Inhibitor, MgCl2, dNTPs and random primers). The reaction proceeded for 10 min at 95 °C, 15 s at 95 °C, 4 min at 60 °C, followed by inactivation at 95 °C for 10 min.

Pre-amplification was done using Human Inflammation PreAmp Primers (Pool A or B) 5 μL, TaqMan PreAmp Master Mix 10 μL, and 5 μL of RT product (cDNA) in a final volume of 50 μL. The temperature sequence was the following: 10 min at 95 °C, 15 s at 95 °C and 4 min at 60 °C (for 14 cycles), followed by inactivation at 95 °C for 10 min. The pre-amplified samples were stored at 4 °C until the PCR was performed.

The TaqMan^®^ OpenArray^®^ Human Inflammation Panel (Applied Biosystems, ThermoFisher Scientific) was used for final PCR detection. These contain TaqMan gene expression assays dried down in 384-well TaqMan Array microfluidic cards targeting pathway relevant gene targets. The panel covers 586 genes that have been studied as targets for a range of inflammatory diseases, plus 21 endogenous control genes.

PreAmp products for each sample were mixed and diluted 1:10 with 0.1× TE pH = 8.0. For each sample, 35 μL of 2× TaqMan^®^ OpenArray^®^ Real-Time PCR Master Mix was mixed with 35 μL mixed, diluted PreAmp. 5 μL of each reaction mixture was pipetted into each of 12 wells on an OpenArray 384-well sample plate. Samples were loaded onto OpenArray^®^ plates using the standard Accufill™ protocol. Amplification was performed according to the protocol established for the TaqMan^®^ OpenArray^®^ Human Inflammation Panel with the QuantStudio™ 12 K Flex Real-time PCR system.

### Data analysis and bioinformatics

The mRNA data comprises cycle threshold (Ct) values from the OpenArray QuantStudio™ 12 K Flex Real-time PCR system, which is converted to gene expression levels for a panel of 607 inflammation-related genes and endogenous control genes. The miRNA data set consists of 754 different miRNAs. Both data sets were processed using the *R* statistical programming environment. Missing data points were assigned ‘undetermined’, indicating sequence abundances below the detection limit of the OpenArray platform. Quantile normalization was applied to data sets to adjust for technical variation and to make data points comparable among each other. Features or targets with greater than 10 undetermined values were removed from the analysis. An additional filtering step was performed to remove features showing little or no variation across samples since these targets with relatively constant Ct levels are less likely to be differentially expressed. This was achieved removing features with an interquartile range of <2. A Mann–Whitney test was performed to compare the samples from patients with elevated and normal ICP. Profiling hundreds of genes at the same time represents a large multiple hypothesis-testing problem from the statistical perspective. While typically this is adjusted by computing false discovery rates (FDR), in this case, however, due to the low number of samples, the multiple testing correction was not applied (since none of the targets showed less than 15% FDR).

Once the set of differentially expressed genes was obtained, functional analyses were generated through the use of QIAGEN’s Ingenuity Pathways Analysis (IPA^®^, QIAGEN Redwood City, www.qiagen.com/ingenuity). First, the predicted targets for the differentially expressed miRNAs were identified using miRNA Filter Analysis, which relies on the Ingenuity knowledge database of reported miRNA targets. This allowed the generation of a comprehensive list of fold-change expression values for miRNA and mRNA, which was then subjected to core analysis.

### Data availability

Raw data supporting the results reported in this article can be found in the NASA Life Sciences Data Archive (https://lsda.jsc.nasa.gov/lsda_home.aspx)

## Electronic supplementary material

Supplementary Table 1
