# Supplementary material for: Inflammatory gene expression signatures in idiopathic intracranial hypertension: possible implications in microgravity-induced ICP elevation
Source: NPJ Microgravity. 2018 Jan 11;4:1. doi: 10.1038/s41526-017-0036-6 (PMC5764966; doi:10.1038/s41526-017-0036-6)
Supplement: Supplementary file 1 — Supplementary Table 1 [file 41526_2017_36_MOESM1_ESM.docx]

| ID# | Gender  Male=1 Female=2 | | Age  (Years) | Weight (kg) | Height (m) | BMI (kg/m2) | Mean ICP (cmH20) | Mean ICP (mmHg) | RNFL OD (µm) | RNFL OD  (µm) | Frisen grade OD | Frisen grade  OS | Signs OD | Signs OS | IHH Yes=1 No=0 |
| --- | --- | --- | --- | --- | --- | --- | --- | --- | --- | --- | --- | --- | --- | --- | --- |
| 001 | | 2 | 40 | 102.3 | 1.6 | 38.7 | 42.7 | 31.4 | 83 | 85 | 1 | 1 | enlarged blind spot | enlarged blind spot | Yes |
| 002 | | 2 | 28 | 96.8 | 1.6 | 37.8 | 19.7 | 14.5 | 94 | 99 | 1 | 1 | scotoma | scotoma | No |
| 003 | | 2 | 51 | 110.0 | 1.6 | 41.6 | 19.2 | 14.1 | 284 | 400 | 2/3 | 4 | constricted visual field | constricted visual field | No |
| 004 | | 2 | 32 | 72.7 | 1.6 | 29.3 | 28.3 | 20.8 | 158 | 135 | 1 | 1 | visual field defect | visual field defect | Yes |
| 005 | | 2 | 30 | 77.7 | 1.7 | 28.5 | 25.5 | 18.8 | 155 | 199 | 1 | 1 | scotoma | scotoma | Yes |
| 006 | | 2 | 32 | 81.8 | 1.6 | 32.0 | 32.5 | 23.9 | 114 | 109 | 0/1 | 0/1 | visual field defect | visual field defect | Yes |
| 007 | | 2 | 28 | 95.9 | 1.8 | 30.3 | 25.5 | 18.8 |  |  |  |  |  |  | Yes |
| 008 | | 1 | 33 | 93.2 | 1.8 | 30.3 | 26.6 | 19.6 | 123 | 112 | 2 | subtle | scotoma | none | Yes |
| 009 | | 2 | 28 | 70.5 | 1.5 | 30.3 | 30.5 | 22.4 | 94 | 95 | 0/1 | 0/1 | visual field defect | visual field defect | Yes |
| 010 | | 2 | 26 | 115.0 | 1.6 | 43.5 | ND | ND | 159 | 168 | 1 | 1 | scotoma | scotoma | ND |
| 011 | | 2 | 18 | 110.5 | 1.7 | 39.3 | 29.6 | 21.8 | ND | ND | ND | ND | ND | ND | Yes |
| 012 | | 2 | 43 | 59.1 | 1.6 | 23.1 | 15.9 | 11.7 | 96 | 98 | 0 | 0 | visual field defect | visual field defect | No |
| 013 | | 2 | 29 | 139.5 | 1.6 | 54.5 | 36.3 | 26.7 | 343 | 576 | 3 | 4 | enlarged blind spot | enlarged blind spot | Yes |
| 014 | | 2 | 21 | 112.3 | 1.7 | 41.2 | 35.8 | 26.3 | 215 | 263 | 1/2 | 1/2 | scotoma | scotoma | Yes |
| 015 | | 2 | 45 | 92.7 | 1.8 | 28.5 | 22.8 | 16.7 | 89 | 85 | 0/1 | 0/1 | scotoma | scotoma | No |
| 016 | | 2 | 47 | 68.1 | 1.6 | 25.8 | 31.7 | 23.3 | 212 | 146 | 3 | 2 | scotoma | scotoma | Yes |
| 017 | | 1 | 32 | 114.5 | 1.8 | 36.2 | 24.3 | 17.9 | 119 | 112 | 2 | 2 | scotoma | scotoma | No |
| 018 | | 2 | 51 | 70.0 | 1.7 | 24.9 | 15.0 | 11.0 | ND | ND | ND | ND | ND | ND | No |
| 019 | | 2 | 32 | 88.6 | 1.6 | 35.2 | 31.8 | 23.4 | 131 | 221 | 1 | 2 | nasal sector defect | nasal sector defect | Yes |
| 020 | | 2 | 28 | 127.27 | 170.00 | 44.0 | 37.33 | 27.46 | 186 | 155 | ND | ND | ND | ND | ND |
| 021 | | 2 | 47 | 80 | 165.00 | 29.4 | 21.83 | 16.06 | ND | ND | ND | ND | ND | ND | ND |
| 022 | | 2 | 23 | 106.82 | 168.00 | 37.8 | 42.17 | 31.02 | 445 | 393 | ND | ND | ND | ND | ND |

**Supplementary Table 1.** Patient demographics and clinical data. Abbreviations: Intracranial pressure (ICP), bone mass index (BMI), average retinal nerve fiber layer (RNFL) measured by optical coherence tomography (OCT). Twenty-two subjects were enrolled in the study, 21 of which had successful LP. Patients were grouped into the normal to mildly elevated ICP (gray text) and patients with high ICP. The cut off between normal-low and elevated ICP was established at 18 mm Hg.
